# The Karyotype of the Yellow Dung Fly, *Scathophaga stercoraria*, a Model Organism in Studies of Sexual Selection

**DOI:** 10.1673/031.010.11801

**Published:** 2010-07-26

**Authors:** Sonja H. Sbilordo, Oliver Y. Martin, Paul I. Ward

**Affiliations:** ^1^ Zoology Museum, University of Zürich, Winterthurerstrasse 190, CH-8057 Zürich, Switzerland; ^2^ Institute for Integrative Biology IBZ, Experimental Ecology, Universitätsstrasse 16, CH-8092 Zürich, Switzerland

**Keywords:** antagonistic, dimorphism, Diptera, heterochromatin, *Scatophaga*, sex chromosome, sexual conflict

## Abstract

Knowledge of karyotypical characteristics of a species is essential for understanding how sexually selected and sexually antagonistic traits evolve. The yellow dung fly *Scathophaga stercoraria* L. (Diptera: Scathophagidae) is an established model system for studies of sexual selection and sexual conflict, but karyotypical data are lacking to date. Here, the karyotype of *S. stercoraria* was characterized using conventional Giemsa-staining and C-banding techniques. The diploid chromosome set consists of 6 pairs of bi-armed meta- or submetacentric chromosomes. The sex chromosomes are the largest chromosomes and constitute 30% of the total length of the diploid set in females and about 25% in males. Males are the heterogametic sex, and the length of the Y chromosome is about three-quarters of that of the X chromosome. C-banding revealed that both sex chromosomes are largely heterochromatic. In contrast, in the five autosome pairs, heterochromatin is limited to narrow bands in the centromeric regions. This karyotypic information will help provide a more profound understanding of the inheritance of phenotypic variation in reproductive traits and the chromosomal locations of underlying genes.

## Introduction

In dioecious animals with genetic sex determination, the chromosome sets of the two sexes differ in the appearance of the sex chromosomes. Depending on the species, either males or females may be the carriers of the heteromorphic sex chromosome pair (Reinhold 1998). In dipteran insects, the most common sex chromosome system is the XX/XY system. Here females possess two equivalent X chromosomes, while males possess an X and a Y chromosome that is usually degenerated and has hence lost much of the genetic activity (Charlesworth 1996; Rice 1996). The primary signals underlying sex determination may be the ratio of X chromosomes to autosomes, as in *Drosophila melanogaster*, for example. In other species, a specific male-determining factor is responsible for sex determination, e.g. as in *Musca domestica* and several *Chrysomya* species. This factor is often but not always located on the Y chromosome (Ullerich 1976; Hediger et al. 1998a; Schütt and Nöthiger 2000; Ullerich and Schöttke 2006).

Sex chromosome heteromorphism has major implications for the inheritance and expression of X linked genes. In males, the homology between the sex chromosomes is usually restricted to a small region. Therefore, the sex chromosomes can recombine in females, but not in males (Charlesworth 1996; Rice 1996). Males are hemizygous for X linked genes, so the amount of gene products of X linked genes have to be equalized in the two sexes. Several mechanisms have evolved to compensate the expression of X-linked genes. In dipteran insects such as *D. melanogaster* or *Sciara ocellaris*, dosage compensation is achieved by up-regulating the transcription rates of the genes on the single X chromosome in males (Ruiz et al. 2000). Alternatively, if the sex chromosomes carry only very few genes, dosage compensation may not be necessary, as found, for example, in *M. domestica* (Schütt and Nöthiger 2000).

There is convincing theoretical and empirical evidence that phenotypic variation in sexually selected and sexually antagonistic traits is caused by Xchromosomal genes (Rice 1984; Reinhold 1998). Examples of X-linked expression of sexually selected traits include sex-limited traits such as the size of the ventral sperm receptacle in females (stalk-eyed fly, *Cyrtodiopsis dalmanni*: Johns and Wilkinson 2007) or sperm length in males (yellow dung fly, *Scathophaga stercoraria*: Ward 2000; stalk-eyed fly, *C. dalmanni*: Johns and Wilkinson 2007), as well as sexually dimorphic traits such as eyespan in the stalk-eyed fly *C. dalmanni* (Wolfenbarger and Wilkinson 2001).

Sex-linked expression of X-linked genes may be commensurate with the size of the X chromosome (Wolfenbarger and Wilkinson 2001). Indeed, the relationship between the relative length of the X chromosome in the set and the effect of X linked genes has previously been used to assess the influence of X chromosomal genes on expression of sexually selected traits (discussed in Wolfenbarger and Wilkinson 2001). Crucially, this appears to be based on the assumption that the gene contents of the X chromosome and the autosomes are similar. However, in many insect species, the X chromosome is largely heterochromatic, e.g. in several *Chrysomya* species and in *M. domestica* (Bedo 1991; Hediger et al 1998b; Ullerich and Schöttke 2006), so relative gene density is in fact probably lower than on euchromatic autosomes, potentially concealing the relative importance of X linked genes. Additionally, dosage compensation by twofold hyperactivation of X-linked genes in males may double the magnitude of Xchromosome effects (Wolfenbarger and Wilkinson 2001). Thus, precise knowledge of karyotypical characteristics of a species, including the distribution of heterochromatin as well as the mechanism of dosage compensation, would be essential for a complete understanding of how phenotypic variation in sexually selected and/or sexually antagonistic traits evolves.

Since the pioneering work of Parker (Parker 1970a, 1970b), the yellow dung fly *Scathophaga stercoraria* L. (Diptera: Scathophagidae) has become a classic model organism for the study of pre- and postcopulatory sexual selection (Simmons 2001; Ward 2007; Bussière et al. 2009) and sexual conflict (Hosken et al. 2001; Martin et al. 2004; reviewed in Parker 2006). A large range of reproductive traits thought to be under sexual selection, such as the reproductive tract of the female (Hosken et al. 1999; Arthur et al. 2008), sperm size (Ward 2000) and sperm utilization patterns (Sbilordo et. al. 2009), have been thoroughly investigated in this taxa. However, in contrast to the genome size (Garner 2002), the chromosomal constitution of this species remains unknown to the authors' knowledge. Here, a detailed description of the karyotype of the yellow dung fly is provided with special reference to the sex chromosome system and the morphology of the sex chromosomes, including the distribution of heterochromatin.

## Materials and Methods

Cytological analyses were carried out either on neural ganglia of larvae or on reproductive tissues of adults. All animals used during this study were derived from laboratory stocks originally established from a population of *S. stercoraria* near Fehraltorf, Switzerland. Animals used were either from the first laboratory generation or derived from parental flies reared for more than one generation in the laboratory. Dung fly larvae and adults were housed in a climate chamber under standard conditions (20° C, 13:11 L:D). The larvae were reared in cow dung and used when they were between five and six days old. After eclosion, the adults were kept in vials (separated by sex, one to three flies per vial) and fed with sugar, water and adult *Drosophila sp. ad libitum*. Chromosome preparations of gonadal tissue were made six to twelve days after eclosion of the adults. Prior to killing, adult flies were cooled to 10° C for two days and then heated under a warm light for about 45 minutes to accelerate cell division (personal communication, M. Hediger-Niessen).

### Conventional Giemsa staining

The preparation of chromosome spreads was carried out according to El Agoze et al. (1992) with slight modifications. Briefly, the neural ganglia of the larvae were dissected in 1% sodium citrate, and the gonads of the adults were dissected in Ringer solution. Both tissues were treated hypotonically in 1% sodium citrate for ten to fifteen minutes. The hypotonic solution contained 0.05% of colchicine to increase the amount of metaphasic cells. After hypotonic treatment, the tissues were fixed in freshly prepared 3:1 ethanol-glacial acetic acid for five minutes. This fixative was replaced immediately before slide preparation. The fixed tissues were minced in 60% acetic acid, and two to four drops of the resulting cell suspension were dropped onto cleaned, ice-cold slides. These slides were then placed on a hot plate at 45° C to dry. During evaporation of the solution, slides were moved gently to distribute the cell suspension evenly over the slide. Slides were then allowed to dry for at least two hours before they were stained with 10% Giemsa in a phosphate buffer solution (pH = 6.8) for four to six hours. After staining, slides were rinsed for one minute with deionized water. Chromosome counts were carried out on 92 well-spread metaphases prepared from the tissues derived from 21 individuals: three larvae, 13 males, and five females. Chromosome lengths were measured on ten good quality mitotic cell spreads from each sex derived from three males and three females, respectively. Measurements were performed on microscope images (100x) conveyed to a computer running the image analysis system KS 300, version 3.0 (Zeiss, www.zeiss.com). The chromosomes were classified morphologically based on the centromeric indices according to Guerra (1986). Digital images of the measured cell spreads were taken for illustration purposes. A multivariate general linear model was performed to compare the length of the two sex chromosomes within each sex. Residuals of the models did not deviate from normality (KolmogorovSmirnov tests: p > 0.42).

### C-banding

Suitable mitotic chromosome spreads were marked on slides freshly stained with Giemsa, and the slides subsequently were destained. Slides were passed through an ascending alcohol series (70% and 99% ethanol), washed in deionized water, and air dried. The C-banding technique (Sumner 1972) was then applied. Slides were treated with 0.2N HCl for 30 min at room temperature and rinsed with deionized water. The slides were then placed in a freshly prepared 5% aqueous solution of Ba(OH)_2_ at 50° C for five minutes and washed with deionized water. After these treatments, slides were allowed to dry completely at room temperature for 48 h. The slides were then incubated in 2xSSC (0.3 *M* NaCl containing 0.03 *M* tri-sodium citrate) for 60 min at 60° C, rinsed with deionized water and stained with 10% Giemsa in phosphate buffer (pH = 6.8) for six hours. After a final washing with bidistilled water, the slides were air-dried and mounted in Eukitt quick-hardening mounting medium (Fluka, www.sigmaaldrich.com). Digital photographs were taken with a Nikon Eclipse E600 microscope (www.nikon.com) fitted with a ProgRES C 5 digital camera (www.progrescamera.com). Measurements were performed on the photograph using the software ImageJ (http://rsbweb.nif.gov/ij/). The schematic drawing of C-banded mitotic chromosomes of males was based on the assessment of banding patterns in 20 metaphase spreads.

**Figure 1.**
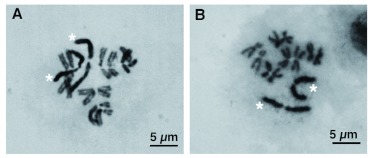
Mitotic cell spreads of a male (A) and a female (B) *Scathophaga stercoraria*. The sex chromosomes marked with asterisks (*) stain more darkly with Giemsa. High quality figures are available online.

## Results

The modal chromosome set of *S. stercoraria* was diploid and consisted of twelve chromosomes, comprising five pairs of autosomes and one pair of sex chromosomes (Figure 1). All chromosomes of the set were biarmed, metacentric, or submetacentric (Table 1).

**Figure 2.**
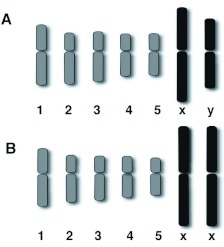
Diagrammatic representation of the Giemsa stained idiogram of the male (A) and the female (B) of *Scathophaga stercoraria*. Strong labeling is depicted in black, weaker labeling in grey. High quality figures are available online.

**Figure 3.**
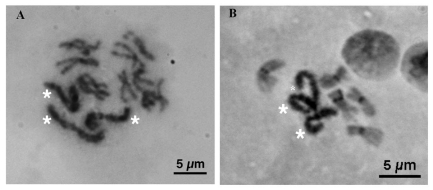
Mitotic cell spreads of the exceptional female *Scathophaga stercoraria* with three sex chromosomes (A) after Giemsa staining and (B) C-banding technique. The sex chromosomes are marked with asterisks. The sex chromosomes are stained over almost their entire length using both procedures. All autosomes showed small dark stained bands in the centromeric region after C-banding (B). High quality figures are available online.

**Figure 4.**
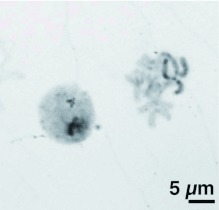
C-banding of a representative mitotic cell spread of a male *Scathophaga stercoraria*. Both sex chromosomes are more darkly stained than the C-band negative regions of the autosomes, and the Y chromosome additionally exhibits extensive darkly stained blocks. The autosomes are C-banding negative except for narrow bands in the centromeric regions. High quality figures are available online.

The sex chromosomes were the two longest chromosomes of the set constituting 30% of the total length of the diploid set in females and 25% in males, respectively (Figure 2, Table 1). Both of the sex chromosomes were stained more intensely by Giemsa than the autosomes (Figures 1, 2, 3). The more intensive staining of sex chromosomes as compared with autosomes was consistent with previous work on other dipterans, e.g. the housefly *M. domestica* (El Agoze et al. 1992). The homologous autosomes were closely associated with each other and arranged in pairs in most of the mitotic cell spreads. This was most probably a result of somatic pairing during mitosis (see Metz 1916). The sex chromosomes were more loosely associated in the spreads (Figures 1, 3, 4). This observation coincides with the observation of mitotic chromosomes from ovarian tissue and somatic cells of females in *M. domestica* (Metz 1916) and the sex chromosomes of *Melophagus ovinus* males (Cooper 1941).

**Table 1.**
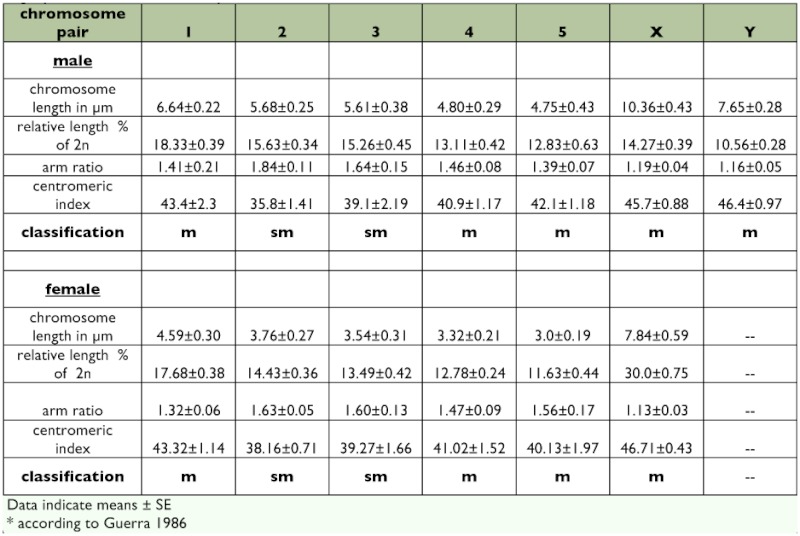
Morphometric traits of the chromosomes based on 10 mitotic plates for each sex. The chromosomes were slightly more condensed in cell spreads of females than of males.

**Figure 5.**
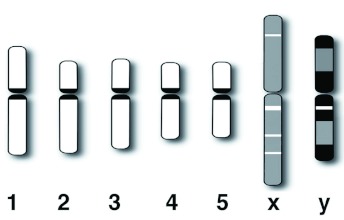
Diagrammatic representation of the C-banding idiogram of a male *Scathophaga stercoraria*. Strongest labeling is depicted in black weaker labeling in grey and C-banding negative regions are depicted in white. High quality figures are available online.

Males are the heterogametic sex in this species because the sex chromosomes were significantly dimorphic in size (GLM absolute length: F_1, 19_ = 27.90, p < 0.001; GLM relative length: F_1, 19_ = 60.82, p < 0.001). In females, sex chromosome length did not differ significantly (GLM absolute length: F_1, 19_ = 0.42, p = 0.525; GLM relative length: F_1, 19_ = 3.815, p = 0.067; (Figures 1, 2). C-banding revealed a predominantly heterochromatic character of both sex chromosomes. C-banding applied to highly condensed metaphase spreads resulted in dark staining of both sex chromosomes over almost their entire length with a poorly resolved banding pattern (Figure 3B). In prometaphase spreads with more extended chromosomes, the sex chromosomes also appeared more strongly stained than the pale C-banding negative parts of the autosomes (Figure 4). In these spreads, the Y chromosome additionally showed a particular staining pattern with large dark staining C-banding positive blocks on either side of the centromeric constriction, as well as on the distal regions of the chromosome arms. A small interstitial C-banding negative region was visible in the large centromeric heterochromatin block of the long arm. In contrast to the Y chromosome, the X chromosome did not show any prominent dark staining blocks, and the banding pattern was less pronounced (Figures 4, 5). However, in most of the preparations, three small interstitial C-band negative regions were visible on the long arm, and one lighter stained band was visible on the short arm (Figures 4, 5). The observed C-banding pattern with dark blocks and less intense background staining was similar to those previously observed on extended sex chromosomes of the screwworm *Chrysomya bezziana* (Bedo 1991). Differently stained bands may reflect intrachromosomal differences in the heterochromatin constitution of the sex chromosomes (see Hediger et al. 1998b). The C-banding pattern of autosomes was limited to a single narrow band in the centromeric region in each of the five autosome pairs, regardless of the degree of chromosome condensation (Figure 3B, 4, 5). These C-bands were not consistently visible on every autosome in all spreads. This was probably due to variation in the staining procedure as has been proposed for other insects (Bedo 1991; El Agoze et al. 1992; Ullerich and Schöttke 2006).

Interestingly, one phenotypic female with fully developed ovaries possessed 3 X chromosomes, an aneuploidy observed in all cell spreads stemming from this female (Figure 3). The absolute length of the three sex chromosomes did not differ significantly (GLM: F2, 26 = 0.44, p = 0.650).

## Discussion

Male heterozygosity for sex chromosomes has been proposed for the yellow dung fly in a previous study (Ward 2000), and this could be confirmed based on the karyological data presented here. *S. stercoraria* has five pairs of autosomes and two sex chromosomes, and there is significant size dimorphism in the sex chromosomes of males. A diploid chromosome complement of 12 chromosomes is concordant with other fly species, e.g. the housefly *M. domestica* (El Agoze et al. 1992), the olive fruit fly *Bactrocerca oleae* (Mavragani-Tsipidou 2002), and several blow fly species e.g. *Chrysomya megacephala* and *C. putoria* (Ullerich 1976; Parise-Maltempi and Avancini 2001), *C. chloropyga, C. varipes* (Ullerich 1976) and *C. bezziana* (Bedo 1991). While all these species have the same chromosome number, there exists striking diversity in the length of the sex chromosomes. In *B. oleae, C. megacephala, C. chloropyga*, and *C. varipes*, the sex chromosomes are the two shortest chromosomes of the complement (Ullerich 1976; Parise-Maltempi and Avancini 2001; Mavragani-Tsipidou 2002). In *M. domestica* and *C. putoria* the X chromosomes are approximately autosomal size while the Y chromosome is the smallest chromosome of the set (El Agoze et al. 1992; Parise-Maltempi and Avancini 2001). In marked contrast to all these species, the karyotypes of *C. bezziana* (Bedo 1991) and *S. stercoraria* are dominated by the sex chromosomes, with both being much larger than the autosomes. These few examples indicate that the phylogenetic relationship between species is a very poor predictor for the relative length of the sex chromosomes within species. Sex chromosomes are thought to evolve very rapidly under antagonistic sexual selection (Van Doom and Kirkpatrick 2007), and it has been proposed that the influence of sex-linked genes on polygenic sexual dimorphic traits is approximately proportional to the length of the X-chromosome (Lande 1987). However, the X chromosomes are partially or largely heterochromatic in all of these species (Ullerich 1976; Bedo 1991; Hediger et. al. 1998b; Parise-Maltempi and Avancini 2001) Therefore, it would be worthwhile to investigate whether observed differences in sex chromosome length between closely related species coincide with divergence in sexually dimorphic traits and/or mating system or whether the length of sex chromosomes solely reflects different proportions of heterochromatin.

One phenotypic female had three sex chromosomes of approximately equal size, which made it reasonable to assume that these three chromosomes were all X chromosomes. Sex chromosome aneuploidies are relatively common in animals, and they most often produce minor abnormalities, although in some cases the carriers are sterile (reviewed in Sumner 2003). Insects with aneuploid sex chromosome complements have been reported for several other Dipteran species e.g. XXY males in *C. chloropyga* and *M. domestica* (Ullerich 1976; Hediger et al. 1998a), XO females in *C. chloropyga* (Ullerich 1976) and an XXX female in *Phormia regina* (Ullerich 1961). The occurrence of aneuploid sex chromosome sets may indicate that sex in the concerned species is not determined by the X:autosome ratio as in *D. melanogaster* (Schutt and Nöthiger 2000), but rather by a male-determining factor (Ullerich 1976).

In conclusion, the evolution of sex chromosomes and their involvement in sexual selection and conflict are highly active fields of research (Arnqvist and Rowe 2005). These rapidly evolving chromosomes are considered to be evolutionary hotspots with a central role in the speciation process (see e.g. Presgraves 2008). A range of phenotypic traits likely to be under sexual selection such as body size (Simmons and Ward 1991) or copulation behavior of males (Mühlhäuser et al. 1996) have already been shown to be partially genetically determined in *S. stercoraria*. Now, with more precise knowledge concerning sex chromosomes, such studies could use this as foundations to provide a more complete picture of sexual selection in this important model organism.

## Abbreviation

GLM: general linear model
